# Glycosylhydrolase genes control respiratory tubes sizes and airway stability

**DOI:** 10.1038/s41598-020-70185-w

**Published:** 2020-08-07

**Authors:** Matthias Behr, Dietmar Riedel

**Affiliations:** 1 Institute for Biology, Leipzig University, Philipp-Rosenthal-Str. 55, 04103 Leipzig, Germany; 2 Max-Planck-Institute for Biophysical Chemistry, Electron Microscopy Group, 37077 Göttingen, Germany

**Keywords:** Cell biology, Developmental biology, Evolution, Genetics, Zoology, Respiration

## Abstract

Tight barriers are crucial for animals. Insect respiratory cells establish barriers through their extracellular matrices. These chitinous-matrices must be soft and flexible to provide ventilation, but also tight enough to allow oxygen flow and protection against dehydration, infections, and environmental stresses. However, genes that control soft, flexible chitin-matrices are poorly known. We investigated the genes of the chitinolytic glycosylhydrolase-family 18 in the tracheal system of *Drosophila melanogaster*. Our findings show that five chitinases and three chitinase-like genes organize the tracheal chitin-cuticles. Most of the chitinases degrade chitin from airway lumina to enable oxygen delivery. They further improve chitin-cuticles to enhance tube stability and integrity against stresses. Unexpectedly, some chitinases also support chitin assembly to expand the tube lumen properly. Moreover, Chitinase2 plays a decisive role in the chitin-cuticle formation that establishes taenidial folds to support tube stability. Chitinase2 is apically enriched on the surface of tracheal cells, where it controls the chitin-matrix architecture independently of other known cuticular proteins or chitinases. We suppose that the principle mechanisms of chitin-cuticle assembly and degradation require a set of critical glycosylhydrolases for flexible and not-flexible cuticles. The same glycosylhydrolases support thick laminar cuticle formation and are evolutionarily conserved among arthropods.

## Introduction

Oxygen uptake is vital for all animals and an essential factor responsible for the limitation of fitness^1^. Arthropods, especially insects, actively perform tracheal ventilation and diffusion to oxygenate their organs^2^. The tracheal system is a hallmark of insects limiting their sizes, even in terms of giant evolutionary insects when hyperoxia occurred during the late Carboniferous and early Permian^3^. The *Drosophila* respiratory system consists of multicellular and unicellular tubes. They together form a complex airway network that transfers oxygen to target tissues^4,5^. Oxygen transport requires barriers to multicellular tracheal tubes. The intercellular diffusion barriers form at the lateral membrane by tight junctions analogous septate junctions (SJs), which prevent the paracellular flow of fluids across the tracheal epithelium into the lumina of the airways^6,7^. Chitin-containing cuticles establish extracellular barriers against infections and to withstand tension forces^8,9^. These include hydrophobic layers facing the lumen, which are impermeable to in- and outflux of fluids^10^. Importantly, tracheal chitin-cuticles assemble to assist tube maturation and partially degrade in subsequent steps to support airway clearance during late embryogenesis^11^. A crucial but less understood aspect is how the assembly and degradation of chitin-cuticles can occur^12^ while maintaining protective barriers and integrity of the tubular system.

Tracheal branching is genetically highly controlled and often evolutionarily conserved^13–17^. After initial branch outgrowth, the tracheal cells produce chitin and secrete chitin-associated proteins on their apical surface^18^. Chitin-synthase complexes synthesize Chitin-nanofibers at the apical cell membrane^19^. These nanofibers integrate into a matrix of chitin-binding proteins, such as Obstructor-A (Obst-A), that organize the chitin-fiber network^8^. The deacetylases Serpentine (Serp) and Vermiform (Verm) further modify the physicochemical properties of chitin-cuticle^20,21^. Other proteins, such as Knickkopf (Knk), may protect the new chitin-cuticles from degradation^22,23^. In late embryos, the chitin-cuticle further establishes an elastic cable-like structure that develops within the tracheal lumen. This chitin-cable provides mechanical tensions that balance other forces to determine the length and diameter of tubes^24,25^. At the end of embryogenesis, tracheal cells need to internalize contents of the chitin-cable, in a process called airway clearance, which subsequently includes initial gas-filling of the branch system^26,27^. In parallel, tubes establish a chitin-cuticle at their apical surface, which forms a waterproof barrier and thus enables gas-transport^28–30^. This late embryonic tracheal cuticle is of crucial importance since any inhibition of tracheal oxygenation in first-instar larvae prevents further body growth and molting into the next larval stages^31,32^.

Very little is known about, if, when, and how the tracheal chitin-matrix is processed or degraded in embryos. A critical mechanism for chitin-matrix degradation must be the enzymatic cleavage of chitin polymers by chitinases. These catalyze the hydrolysis of glycosidic bonds in chitin polymers. Chitinases belong to the conserved glycosylhydrolase family 18, widespread in the animal kingdom^33–36^. The human glycosylhydrolases act in lung epithelial cells, macrophages, and eosinophilic cells to inhibit chitin-induced inflammations^37,38^. A common characteristic of all members of the glycosylhydrolase family 18 is the presence of one or more putative catalytic domains, represented by the Glyco18 domain. In silico analysis revealed a large number of glycosylhydrolase family 18 members in insects. Similar to humans, insects possess enzymatically active Chitinases and inactive Chitinase-like proteins. The latter are known as Imaginal disk growth factors (Idgf)^34,39,40^. The analysis of the amino acid sequences of the catalytic domains led to the identification of the genes coding for the glycosylhydrolases family 18 members in many various insects. The *Drosophila* family comprises sixteen members grouped into ten Chitinases (Cht2, Cht4-12), with a single gene encoding for Cht1, Cht3 and Cht10, and six Idgfs (Idgf1-6)^34,41,42^.

Individual domain arrangement, which includes signal-peptides, catalytic-, and transmembrane-domains and chitin-binding domains, may cause functional differences among insect glycosylhydrolases family 18. Thus, members can be classified into eight groups^34^: The *Drosophila* Chitinases contain single (Cht2,4,5,6,8,9,11,12) or more (Cht7, Cht1/Cht3/Cht10) catalytic Glyco18 domains, some possess a chitin-binding domain (Cht5,7,12) others a transmembrane domain (Cht7). In contrast, as Idgf proteins (group V) contain one Glyco18 domain, which lacks a critical glutamate residue, they do not possess chitinolytic activity^40^. Except for Chitinase11, *Drosophila* glycosylhydrolase members possess a signal peptide suggesting that they are all secreted^42^. Many of the enzymes may catalyze the turnover of old cuticles during molting. However, with few exceptions, the functional role of most chitinases and chitinase-like proteins in insect development is poorly understood.

Previously, we showed that a set of chitinases (*cht2, cht5, cht7, cht9, cht12*) and most *idgf* genes are required for supporting the barrier function of tight lamellar epidermal cuticles during larval development^42^. However, whether Chts and Idgfs support the formation and degradation of the tracheal soft and non-laminar cuticle is not known. We carried out the first systematic tracheal specific knockdown studies of *Drosophila* glycosylhydrolase family 18 members. Our studies identified the genes that are essential for the formation and function of the tracheal respiratory tract. We show that chitinases *cht 2, cht5, cht6, cht7*, and *cht12* and imaginal disc growth factors *idgf1, idgf3*, and *idgf6* are crucial for airway integrity. These chitinases strengthen the supporting function of the chitin-cuticles against mechanical stress, with the consequence of unstable airways in knockdown embryos and larvae. Some chitinases and idgfs are involved in tube size control of primary branches and also in airway clearance of the chitin-cable. Importantly, almost the same *chts* and *idgfs* are also involved in the formation of laminar epidermal thick and laminar larval cuticles^42^. Interestingly, the identified chitinases contain single orthologues among insects, including mosquitos^41^. Our study identifies eight genes of the glycosylhydrolases family 18 that are essential for the assembly and degradation of the barrier-forming chitin-cuticles, both laminar and non-laminar, and thus for establishing different kinds of cuticles in insects.

## Results

In previous publications, we showed the expression of some chitinases and idgf genes in cuticle forming organs, which include the tracheal system^42,43^. However, the expression pattern of other glycosylhydrolase families 18 genes was still unclear. To access systematically which *chitinase* and *idgf* genes might be involved in the tracheal chitin-matrix function, we supplemented our initial analyses and further performed whole-mount RNA in situ hybridizations. We further compared our data with those from fly-databases [Fly Express & BDGP in situ homepage]. Collectively, the identified gene expression patterns led to the following potential tracheal candidates: *cht2, cht3 (the single cht3 gene also includes cht1 and cht10 genes), cht5, cht6, cht7,* and *cht12* genes are expressed from embryogenesis onwards in the tracheal cells (summarized in Fig. S1). We could not detect any tracheal expression of genes *cht4, cht8, cht9*, *cht11*, *idgf2*, *idgf4,* and *idgf5* in embryos.

**Figure 1:**
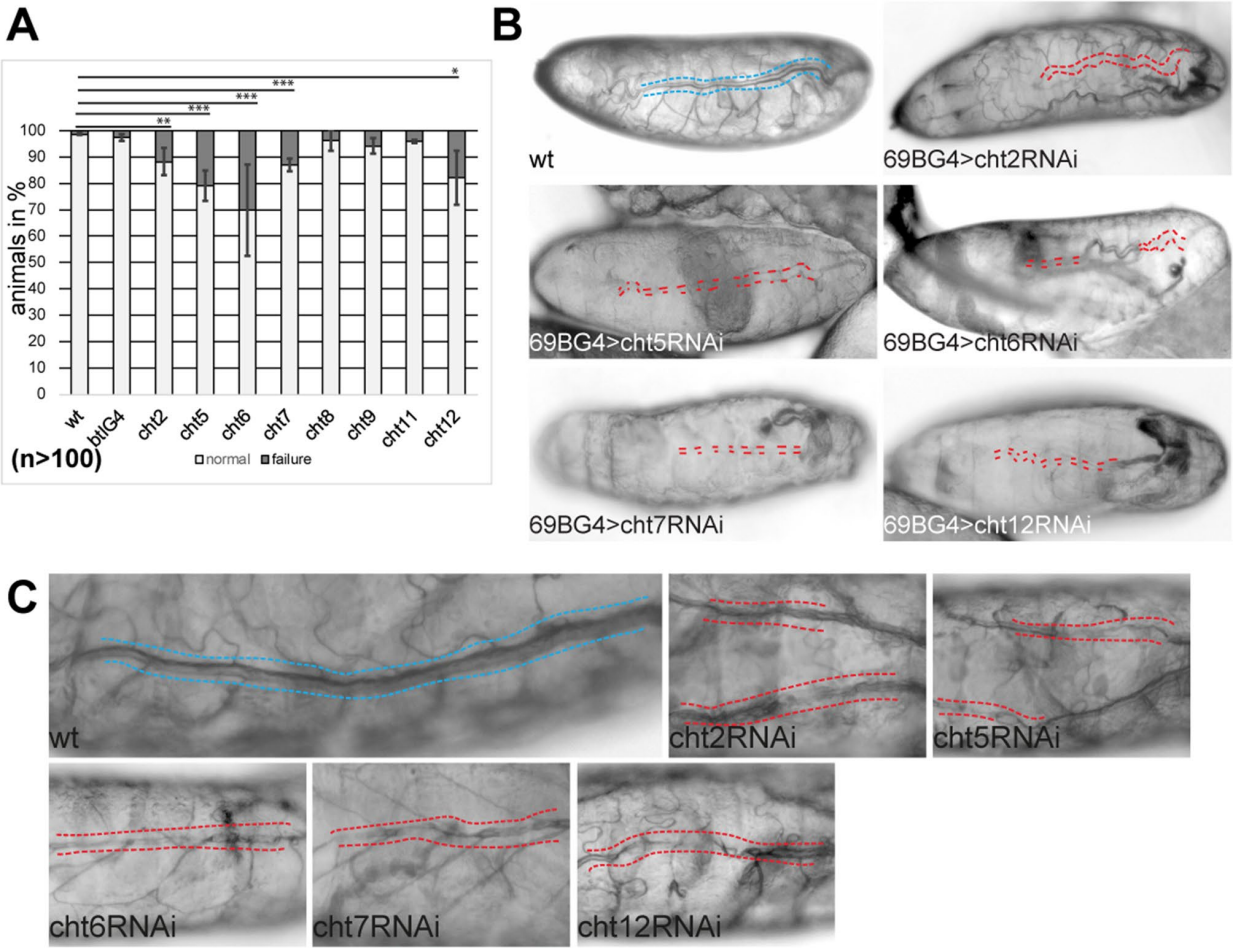
Reduced RNA levels of chitinases lead to tracheal defects. (**A**) The knockdown of *Drosophila chitinase* genes with the tracheal btl-Gal4 driver lines caused tracheal gas-filling defects. We observed variable gas filling phenotypes from complete loss to partial failure in late embryos and freshly hatched larvae. (**B**) We observed gas filling defects upon knockdown with the ectodermal 69B-Gal4 driver line, which is active in the tracheal cells. Tracheal gas-filling defects are represented by dark grey bars (**A**) and by red dashed lines (**B**), while blue lines show normally aerated airways (**B**). Shown are representative images of phenotypes. Error bars represent standard errors; p-values are represented by asterisks: *p < 0.05, **p < 0.01, ***p < 0.001. (**C**) The tracheal expression of *cht2,5,6,7,* and *cht12* RNAi caused twisted, crushed, and discontinuous tubes (red dashes) in first instar larvae. The wt showed normal tubes and gas-filling (blue dashes). Images of in vivo brightfield microscopy.

Next, we examined whether tracheal expression of chitinases is essential for tracheal oxygen transport function. Tracheal tubes form during the second half of embryogenesis. The initial gas filling of airways occurs at the end of embryogenesis at stage 17. Previous publications showed that gas filling defects are not lethal to the embryos, but remain even after hatching as first instar larvae^28,29^. Nevertheless, embryonic gas filling defects could be an indicator of an irregular chitin-matrix. To get a first overview of chitinases that might play a role in tracheal development, we analyzed the gas filling in vivo in late embryos and first instar larvae. For this purpose, we carried out gene-specific knockdown analyses. In a previous publication, we demonstrated the high knockdown efficiency of UAS-*cht* RNAi (Supplementary Table) upon ubiquitous RNAi expression RNA levels of the respective genes reduced below 20% of the wild-type level^42^. To obtain a tracheal specific knockdown of the candidate genes, we combined flies of the UAS-*cht* RNAi lines with the tracheal-specific Gal4 driver line breathless-Gal4 (btl-G4). Btl-G4 expresses Gal4 in tracheal cells throughout development^44^ and has been used successfully to study airway gas-filling^45,46^. These experiments led to a tracheal-specific knockdown of the individual candidate genes in the progeny embryos and larvae. For all following tracheal-specific knockdown experiments, we used the genetics above-described unless otherwise indicated. We examined living wild-type (wt), control (driver-line), and knockdown embryos and larvae by brightfield microscopy. More than 98% of the embryos of the wt and control lines completed gas filling. Similarly, in the case of tracheal expression of RNAi of the *cht8, cht9,* and *cht11* genes, more than 95% of the embryos showed regular gas filling. In contrast, we observed respiratory gas filling defects in a significant number of up to 30% of the embryos (n > 100 embryos) upon RNAi based tracheal knockdown of the *cht2, cht5, cht6, cht7, cht12* genes (Fig. 1A). As residual gene activity can be expected^42^, phenotypes of the btlGal4 driven knockdown animals, varied in penetrance, which is evident in the gas-filling statistic and the images. Therefore we examined whether we obtain similar results with another Gal4 line. For this purpose, we repeated the knockdown studies with the 69B-Gal4 line. This line is active in embryonic and larval tracheal cells and also in other ectodermal tissues. With the driver line 69B-Gal4, we observed comparable gas filling defects in embryos and larvae due to RNAi-based knockdown of the genes *cht2, cht5, cht6, cht7, cht12* (Fig. 1B). Thus, our knockdown experiments using the individual UAS-*cht* lines and both Gal4 lines, which are active in the tracheal cells, led to the identification of chitinase genes involved in the tracheal functions.

Next, we were curious if the tracheal chitinases support the function of the tracheal chitinous cuticle. One key function of the tracheal cuticles is the protection of tubes against mechanical stresses. Assuming that chitin-modifications support chitin-cuticle integrity, defects in this could lead to loss of branch stability. To address this, we studied the tracheal branches of knockdown larvae in vivo using brightfield-microscopy. In contrast to wt, the tracheal specific RNAi expression of *cht2,5,6,7*,*12* genes showed twisted, crushed, and discontinuous tubes in first instar larvae upon gene knockdown (Figs. 1C; S2). Thus, our results suggest that *cht2, cht5, cht6, cht7,* and *cht12* chitinases are involved in tracheal tube integrity and tube lumen stability of first instar larvae.

**Figure 2:**
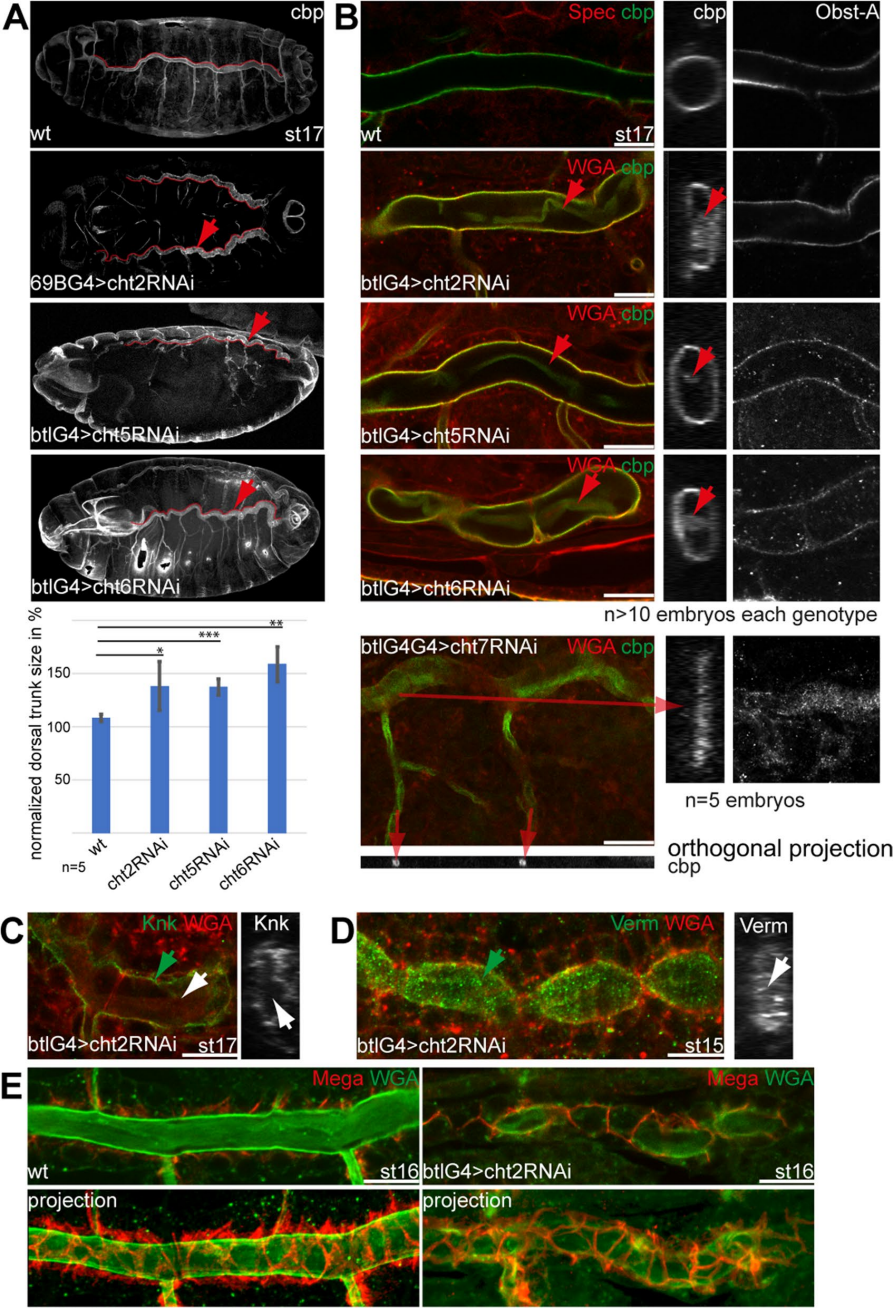
The knockdown of chitinases causes irregular shape of tubes and airway clearance defects. (**A**) Maximum intensity projections of Chitin stainings (cbp) of early stage 17 whole-mount embryos. Upon knockdown of *cht2*, *cht5*, *cht6,* dorsal tracheal trunks (red lines) showed sinusoidal appearance (arrowheads in **A**). All analyzed embryos (n > 15 for each genotype) displayed a curly branch phenotype, but to variable extend. The length of the dorsal trunks of *wt*, *cht2*, *cht5* and *cht6* tracheal (btlG4) knockdown mutants were measured and normalized to corresponding distances. The results are shown in percentages. Error bars represent standard errors; p-values are represented by asterisks: *p < 0.05, **p < 0.01, ***p < 0.001. (**B**) Confocal images show the contents of the chitin-cable (red arrowheads) in Z-stack projections (left) and orthogonal sections (middle panel). The knockdown of *cht2*, *cht5*, *cht6, cht7* failed to remove luminal chitin in stage 17 embryos (red arrows point to chitin residuals). Note the irregular tube lumen in *cht7* knockdown embryo (long red arrow). Unlike chitin, knockdown embryos removed Obst-A from tracheal lumina (Z-stack projections in the right panel). The number of analyzed embryos of each genotype is > 10 per genotype and n = 5 for *cht7*. (**C**) As found in wt (see Fig. S4), stage 17 *cht2* knockdown embryos (n = 14) Knk stainings localize on the apical cell surface (green arrow). The *cht2* knockdown embryos clear the chitin-cable associated Knk (white arrows point to lumen) as also indicated by the orthogonal projection (small image, white arrow). (**D**) The stage 15 *cht2* knockdown embryos (n = 5) show normal Verm distribution within the tracheal lumen (green arrow), as also detected in the orthogonal projection (white arrow point to lumen). Note the sausage-link phenotype in stage 15 *cht2* knockdown embryos, known from apical expansion defects^15^. (**E**) The septate junction core component Mega shows a wt-like distribution in *cht2* knockdown mutants (n = 5). The upper panel shows confocal images, and lower reveals maximum intensity projections. Scale bars indicate 10 µm.

Before the animals establish gas filling and tracheal cuticle integrity, they need to adjust tube lengths and lumen diameter to individual body size during stage 15 and stage 16 of embryogenesis. Tracheal cells massively secrete chitin, which, together with associated cuticle proteins, forms a matrix at the apical cell surface and an intraluminal chitin-cable. Both apical chitin-matrix and intraluminal chitin-cable are essential to control uniform lumen expansion and tube lengths^47,48^. To further investigate which of the tracheal chitinases play a role in tube size expansion, we analyzed chitin in the trachea by using the Alexa488-linked chitin-binding probe (cbp). Besides, we used the Alexa633-linked wheat germ agglutinin (WGA), which is a lectin that detects sugar residues and therefore labels both chitinous structures and surfaces of tracheal cell membranes^8,9^. The tracheal-specific knockdown of *cht2*, *cht5*, *cht6* genes, but not of *cht7* and *cht12*, resulted in tubes with a sinusoidal appearance in the early stage of 17 embryos (Figs. 2A; S3), which is characteristic for oversized tubes^47^. We measured the size of the dorsal trunks, normalized and compared average values with the wt. This revealed a significant enlargement of the tubes by up to 27%, 26% and 46% in *cht2, cht5* and *cht6* knockdown embryos, respectively (Fig. 2A). Thus, our data suggest that tube size expansion involves at least these three chitinases. Defective chitin synthesis and mislocalized chitin-matrix proteins can cause oversized tubes^18,49^. Therefore, we addressed known chitin-cuticle proteins and found that chitinases are not involved in the secretion or localization of those. Knickkopf (Knk,), Obstructor-A (Obst-A) or Vermiform (Verm) appeared wt-like in the tracheal *cht* knockdown mutants (Figs. 2B–D; S4). Further, we found that cell polarity and septate junction markers were not affected upon reduced expression of *cht* genes. The polarity protein Crumbs, as well as Megatrachea (Mega), a claudin-like protein responsible for barrier-forming septate junctions, were typically localized at the apical-lateral and lateral membrane (Fig. 2E, see below). Together, these data indicate that *cht2,cht5* and *cht6* are involved in tube size control, but probably not by modulating the localization of other tracheal cuticle proteins^40^.

After tube length determination has been completed, tracheal cells of embryos of stage 17 internalize the intraluminal chitin-cable contents^27,28,46^. This process requires the DnaJ domain protein Wurst for Clathrin heavy chain recruitment and the clathrin-mediated endocytosis of chitin-associated proteins^9,26,46^. In endocytosis mutant embryos, tubes do not remove extracellular proteins, such as Obst-A, Verm, Knk as well as chitin, from the lumen^9,26,27^. When expressing tracheal-specific RNAi of the *cht2, cht5,* and *cht6* genes in embryos, we did not observe any luminal residues of Knk, Obst-A or Verm at the end of stage 17 as found in *wurst* or *clathrin* mutants (Figs. 2B,C; S4). We assume that the internalization of chitin most likely requires additional enzymatic degradation of chitin polymers or fibers. Therefore, we addressed whether tracheal chitinases may support chitin-cable degradation. Our confocal Z-stacks and orthogonal projections showed that residues of large chitin molecules clogged parts of the central dorsal airways, the so-called dorsal trunks, of btl-Gal4 driven tracheal *cht2*, *cht5,* and *cht6* knockdown embryos at stage 17 (Fig. 2B). Since we analyzed knockdown embryos with slight residual gene activity^42^, the airway clearance phenotype was variable in its strength of appearance as it was the case with the above-shown gas filling defects. Such clogged airways suggest that the *cht2,cht5 and cht6* genes are involved in the degradation of chitin molecules from large airways and that this process is independent of the internalization of proteins. Interestingly, *cht7* knockdown showed residues specifically in the small lateral branches, while the dorsal trunk shape appeared irregular (Fig. 2B).

**Figure 3:**
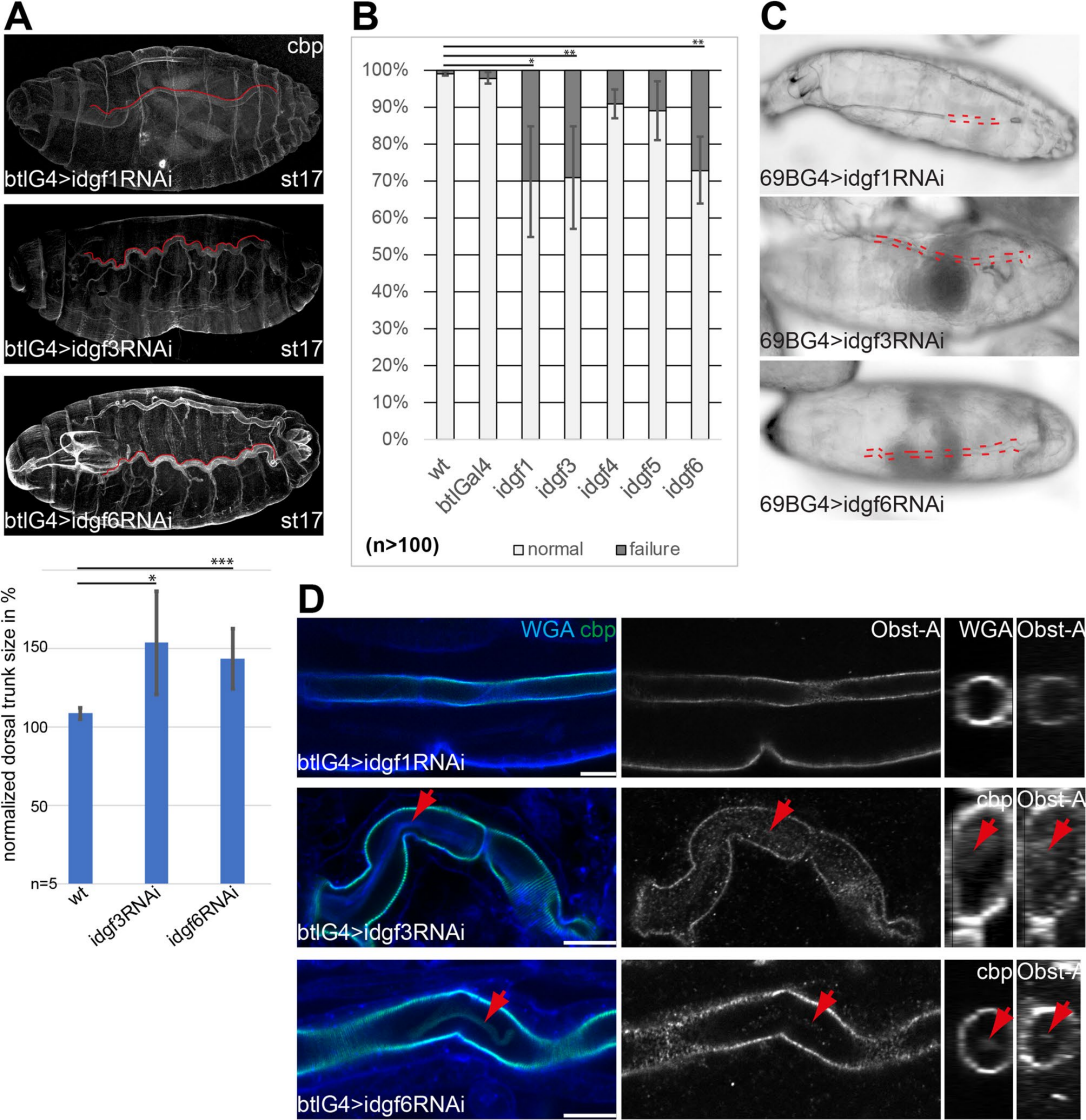
Impairment of *idgfs* leads to tracheal defects. (**A**) Unlike in wt, tracheal specific knockdown of *idgf1*, *idgf3* and *idgf6* caused sinusoidal appearances of tubes (red lines) in early stage 17 embryos (n > 15 each genotype) to variable extend. The length of the dorsal trunks of *wt*, *idgf3* and *idgf6* tracheal (btlG4) knockdown mutants were measured and normalized. The results are shown in percentages. Error bars represent standard errors; p-values are represented by asterisks: *p < 0.05, **p < 0.01, ***p < 0.001. (**B**,**C**) Tracheal gas-filling defects caused by knockdown of *idgfs* with the tracheal btl-Gal4 (**A**) and the ectodermal 69B-Gal4 (**B**) driver lines. Complete failure and also a partial failure of gas filling displayed in late embryos and freshly hatched larvae. Tracheal gas-filling defects are represented by grey bars (**A**) and by red dashed lines (**B**). For control, see Fig. 1. Error bars represent standard errors; p-values are represented by asterisks: *p < 0.05, **p < 0.01, ***p < 0.001. (**D**) The tracheal knockdown of *idgf3* and *idgf6* impaired airway clearance of chitin and Obst-A (arrows in **D**), as shown by Z-stack projections (left and middle) and orthogonal analysis (left panels). Scale bars indicate 10 µm.

The *Drosophila* glycosylhydrolase family 18 also contains six catalytically inactive Chitinase-like Idgf proteins^40^. While recent data documented the need for *idgf* genes for larval epidermal cuticle formation^42^, their role in tracheal chitin-cuticle formation remains elusive. Previously we have shown the high knockdown efficiency that sharply reduced expression levels of *idgf* genes when using UAS-*idgf* RNAi lines^42^ (Supplemental Table). We showed that tracheal cells of late embryos express the *idgf1,idgf3*, *idgf6* genes (Fig. S1)^42^. We knocked-down the three *idgf* genes, and it turned out that the tracheal specific btl-Gal4 driven *idgf3* and *idgf6* RNAi expression led to sinusoidal tube appearance, known from oversized tubes (Fig. 3A). For better comparison, we measured the size of the dorsal trunks, normalized and compared average values with the wt. This revealed a significant enlargement of the tubes by up to 41% and 32% in *idgf3,* and *idgf6* knockdown embryos, respectively (Fig. 3A). The tracheal RNAi expression of *idgf1,3*,*6* but not *idgf 4,5* genes also led to gas filling defects in stage 17 embryos (n > 100 embryos) (Fig. 3B). We repeated the results using the 69B-Gal4 driver line, which led to partial or complete gas-filling defects in the airways of knockdown embryos (Fig. 3C), too. Next, we investigated if *idgfs* are involved in the removal of the chitin-cable from the tracheal tube lumen. Importantly and in contrast to chitinases, the reduced expression of *idgf* genes led not only to residues of chitin but also of extracellular proteins (Fig. 3D). Thus, our data indicate that Idgfs affect tracheal chitin-cuticles via other mechanisms as Chitinases. Nevertheless, the consequences are the same, leading to the airway gas-filling, tubes size, and chitin-cable internalization defects.

**Figure 4:**
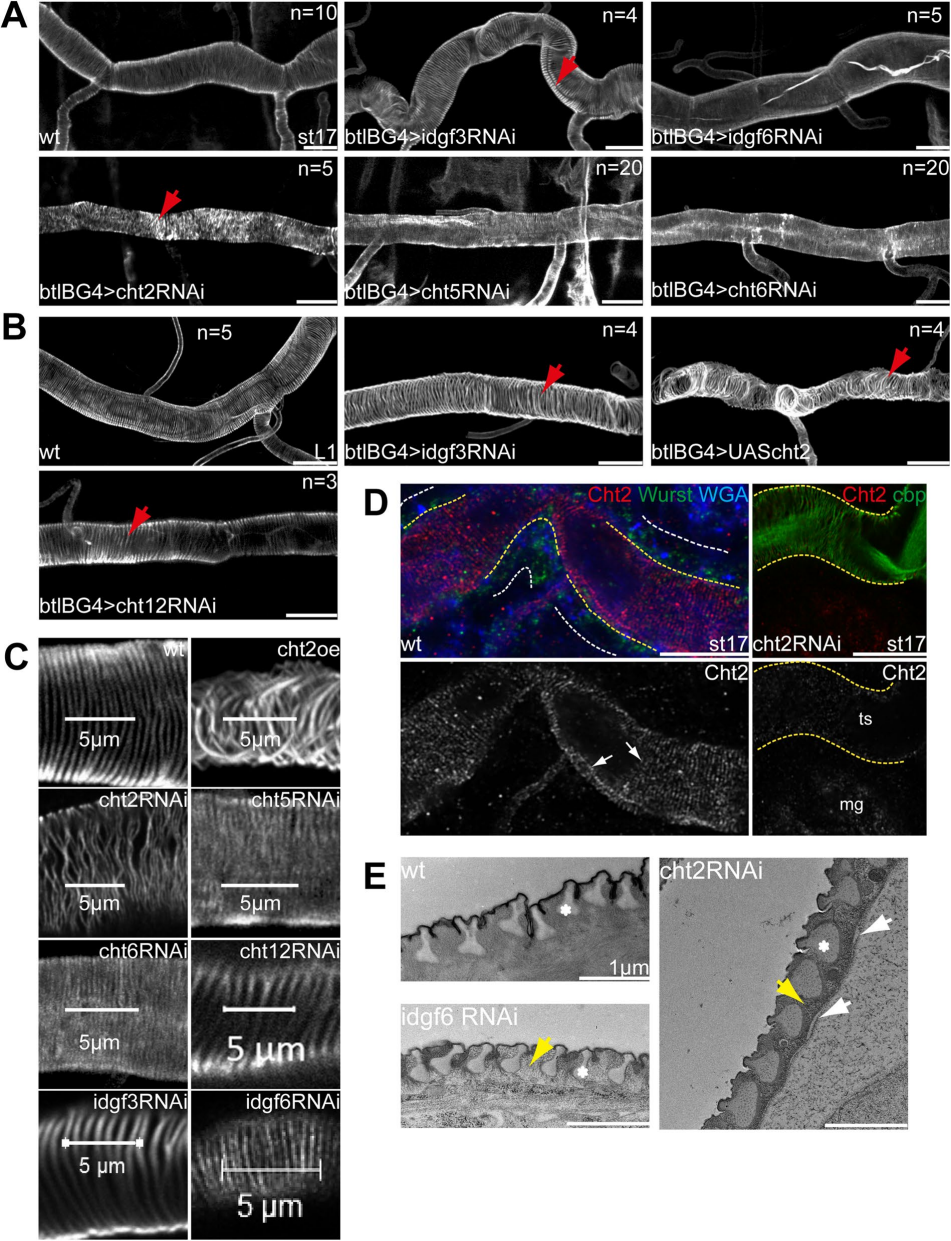
Cht2 is essential for taenidial fold arrangement in embryos and larvae. (**A**,**B**) wt embryos show regular patterns of taenidial folds in stage 17 embryos. In contrast, ectopic expression of *cht2* and knockdown of *cht2* caused irregular taenidial fold arrangement. The knockdown of other tracheal *chitinases* and *idgfs* that showed airway clearance defects did not show improper taenidial fold organization. Note the immense distance between taenidial folds upon knockdown of *cht12* and *idgf3*. Numbers of analyzed animals are indicated in the images. Scale bars represent 10 µm. (**C**) Magnifications of taenidial fold arrangement of wt and knockdown animals. For comparison of taenidial fold distances, 5 µm bars are indicated in the images. (**D**) Airyscan images revealed a taenidial-fold like Cht2 (red) distribution (arrows) at the apical surface of the trachea of stage 17 wt embryos. Wurst (green) marks tracheal cells and WGA (blue) cell membranes. The apical cell surfaces are indicated with yellow dashes and basal surfaces with white dashes. The taenidial-fold like Cht2 staining (red) is reduced upon tracheal *cht2* knockdown. Cbp marks chitin; ts, tracheal system; mg, midgut. Single channels indicate Cht2 staining in grey; scale bar 10 µm. (**E**) Ultrastructure analysis of the tracheal chitin-cuticle in a third instar larva. The wt larvae show a regular pattern of taenidial folds (asterisk) surrounded by filamentous chitin. In tracheal *cht2* knockdown larvae, chitin was grainy (yellow arrow points to chitin), taenidial folds appeared swollen (asterisk), and gaps (arrows) were visible between the cuticle and cell layer. In contrast, tracheal *idgf6* knockdown showed the expected regular wt-like taenidial-fold (asterisk) pattern, but the appearance of the chitin appeared disturbed (yellow arrow point to chitin). Scale bars indicate 1 µm.

Embryonic tracheal cells establish chitin ridges at their apical surfaces, so-called taenidial folds which run perpendicularly along the branch lumen in a helical pattern. They ensure tube integrity in larval airways without compromising the flexibility. Taenidial folds start to develop in embryos at stage 16 and reach their mature form in stage 17 when the tracheal cells remove the chitin-cable from the branch lumen^50^. However, it is not understood whether the formation of such chitin-ridge structures requires chitinolytic manipulations for proper assembly. To address this, we studied taenidial folds in knockdown embryos and first instar larvae. Strikingly, the knockdown of the *cht2* gene led to the disruption of a regular taenidial folds pattern. They appeared loose and irregular in late stage17 *cht2* knockdown embryos and early first instar larvae (Fig. 4A). This early phenotype indicates a defect already during the formation of taenidial folds, suggesting that Cht2 activity is required to assemble these chitin-cuticle structures. To further verify this specific role, we overexpressed *cht2* in the tracheal cells. Higher Cht2 levels did not reduce the luminal chitin, as found in earlier tracheal development^8^, but led to an irregular taenidial fold architecture in embryos, too (Fig. 4B,C). These findings suggest that down and upregulation of Cht2 levels are critical to the proper taenidial fold assembly in stage 17 embryos. In agreement with this, Cht2 showed a filigree taenidial like organization at the apical cell surface of stage 17 wild-type embryos in airyscan images. Only minor Cht2 taenidial-like Cht2 staining was observed upon tracheal *cht2* knockdown (Fig. 4D), which is in line with previous findings that the used RNAi construct lowers RNA levels below 20% of normal expression^42^. Furthermore, Cht2 expression pattern in tracheal chitin-matrices in embryos and larvae and Cht2 protein reduction upon tracheal specific *cht2* knockdown (Figs. S5,S6), supports the multiple tracheal phenotypes found in the knockdown animals. Interestingly, tracheal *idgf3* and *cht12* knockdown embryos and first instar larvae showed a regular helical pattern, but irregular distances between taenidial folds in first instar larvae (Fig. 4B,C). Such potential modulations of taenidial folds in larvae could play a role in tube elongation upon adaption to increasing body size^51^.

During larval development, the molting of the tracheal cuticle takes place. However, it is still unclear whether *chitinases* and idgf genes could also play a role in this process. The tracheal knockdown of the *cht2* gene led to the most severe cuticle defects in early larvae (Fig. 4A–C). Therefore, we investigated the tracheal cuticle of third instar larvae upon tracheal *cht2* knockdowns and for comparison tracheal *idgf6* knockdowns. Since up to 30% of the *cht2* and *idgf6* RNAi knockdown larvae showed lethal gas-filling defects (Figs. 1A, 3A), while others reached the third instar stage, we were able to analyze the tracheal chitin cuticle in late larvae. Our ultrastructural analysis of *cht2* knockdown larvae showed a disturbance of the chitin-cuticle arrangement in third instar larvae. Taenidial folds appeared swollen compared to wt (Fig. 4E). The tracheal chitinous cuticle was not filamentous as in wt but appeared grainy upon tracheal *cht2* knockdown, and separations between the cell and cuticle layers were detectable. The tracheal *idgf6* knockdown led to a non-filamentous chitin pattern, but taenidial-folds were wt-like (Fig. 4E). Accordingly, Cht2 and Idgf6 are essential for the tracheal chitin-cuticle also after cuticle molting.

**Figure 5:**
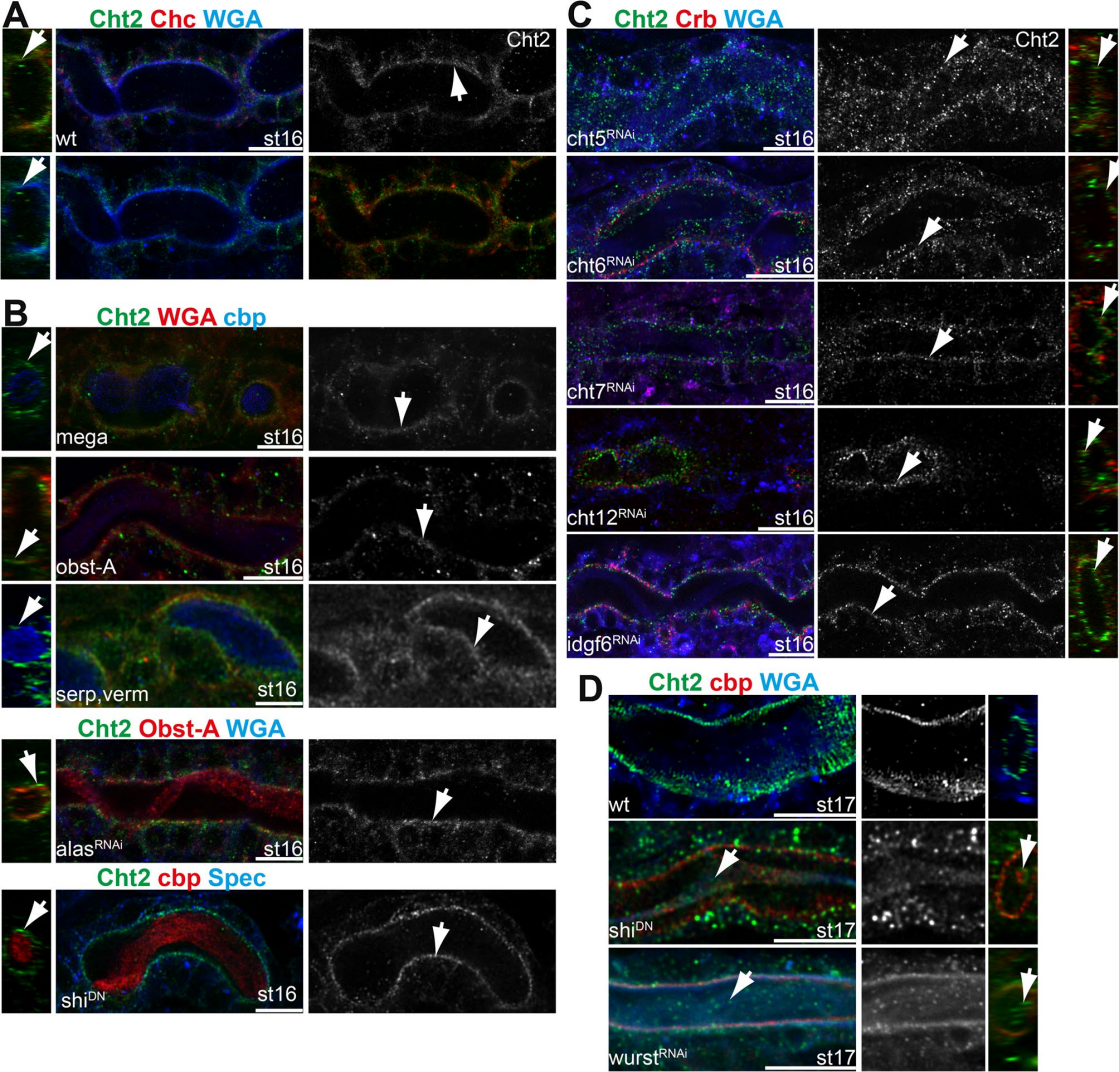
Cht2 accumulates on the apical cell surface. We compared Cht2 (green) staining with the apical cell surface marker WGA, the membrane-skeleton marker α-Spectrin (Spec), the apical polarity marker Crumbs (Crb), the chitin marker cbp and the cuticle protein Obst-A. Single Cht2 channels are shown in grey. (**A**) Confocal images show Cht2 enrichment at the apical cell surface (arrow) of tracheal tubes (see also Fig. S5). (**B**) Wt-like apical Cht2 localization was detectable in stage 16 embryos in *mega*, *obst-A* null-, and *serp-verm* double mutants, upon tracheal RNAi knockdown of *alas*, and *wurst* and by expression of a dominant-negative form of *Drosophila* dynamin gene *shibire* (*shi*). (**C**) The knockdown of *cht5,6,7* and *idgf6* did not disturb apical Cht2 accumulation on the tracheal cell surface. (**D**) The *wurst* and *shi* mutants block endocytosis, which caused unusual Cht2 accumulation within the residual chitin-cable in stage 17 embryos (arrow in **D**) and on the apical surface in stage 16 embryos (white arrow in **B**). The right panel in **D** shows orthogonal projections of the tube lumen. Wt stage 17 embryos do not contain residual chitin-cable and related Cht2 accumulation. For all genotypes, we analyzed n > 7 embryos. Scale bars indicate 10 µm.

One could assume that tracheal cells secrete chitinases mainly to degrade chitin for embryonic airway clearance and larval cuticle molting. In contrast, we show that Cht2 is involved in the assembly of the tracheal chitin-cuticles. Previously, we generated an antibody that specifically recognizes the Cht2 protein in *Drosophila* embryos and larvae and identified its developmental expression pattern in various cuticle-forming organs^43^. However, the tracheal expression pattern and subcellular localization of the Cht2 protein are not known. We found tracheal Cht2 staining in embryos from stage 14 up to stage 16 and observed the tracheal distribution of Cht2, both intracellular and extracellular (Fig. S5). In contrast, intra- and extracellular Cht2 staining in the trachea was strongly reduced in embryos of stage 16 and stage 17 upon tracheal-specific *cht2* knockdown, while Cht2 staining was wt-like in the adjacent epidermis (Fig. S6). Analysis of confocal Z-stacks and orthogonal projections further showed that Cht2 accumulates in the tracheal lumen on the apical cell surface of stage 16 and stage 17 embryos. This apical Cht2 accumulation is evident by colocalization with the cell surface marker WGA and with the chitin marker cbp. It is furthermore apparent with the apically enriched Clathrin heavy chain (Chc) and the apico-lateral cell membrane marker Crumbs (Figs. 4D, 5A,C; S5,S6). Interestingly, tracheal specific *cht2* RNAi knockdown reduced apical Cht2 accumulation when compared to the stainings in the epidermis but did not change chitin staining in the tracheal lumen (Fig. S6A–C). These results show that Cht2 is present on the surfaces of tracheal cells, possibly to support chitin-cuticular assembly in embryos for determining tube sizes before the chitin-cable is degraded at stage 17.

Chitin-matrix assembly occurs at the apical cell surface in a narrow region called the cuticle assembly zone, also known as the Schmidt layer^12^. The localization of proteins within the cuticle assembly zone depends on the following factors. First, septate junctions are involved in the apical secretion of chitin-associated proteins^21,52^. Second, the precise localization of extracellular chitin-proteins and deacetylases depends on the chitin-binding protein Obst-A, which localizes in the assembly zone^8,53^. Third, deacetylases such as Serp and Verm start to modulate chitin cuticle properties in the assembly zone^53^. Fourth, proteins such as Alas play a role in the formation of the barrier-forming cuticle dityrosine network^29^. Loss or reduced function of the above genes affects the tracheal cuticles and some also the extracellular apical distribution of cuticle proteins^8,21,23,29^. However, when analyzing mutant embryos of all the corresponding genes by confocal Z-stacks and orthogonal projections, we found that Cht2 still accumulated apically at tracheal cells (Figs. 5B; S7). Such an apical localization indicates that Cht2 could act independently or upstream of so far known chitin-cuticle modifications mechanisms. Therefore, we next assumed that Cht2 localization in the chitin-cuticle could depend on the other tracheal Chitinases. However, corresponding knockdown embryos, which displayed tracheal defects, did not show disturbance of apical Cht2 accumulation at the cell surfaces (Figs. 5C; S7). Nevertheless, Cht2 localization depends on endocytosis. Blocking early endocytosis in *shibire* (*Drosophila dynamin*) and *wurst* mutants caused Cht2 accumulation at the apical cell surface of stage 16 embryos and in the chitin-cable of stage 17 embryos, which was not the case in stage 17 wt embryos or other analyzed mutants (Figs. 5D; S7). This supports our hypothesis that Cht2 can be involved in tracheal chitin-cuticle assembly and chitin-cable degradation.

To summarize our findings, due to cuticle phenotypes, expression patterns, and localization data, we show that Cht2, Cht5, Cht6, Cht7, Cht12, and Idgf1, Idgf3, Idgf6 act at the flexible tracheal chitin-cuticle to support tube function. Yet, we do not know their molecular roles at the cuticle. Idgfs contain non-chitinolytic domains^40^, suggesting that they support but do not directly degrade chitin. Chitinases contain different protein domains, suggesting that they can perform specific functions (Fig. S1B). All tracheal chitinases have signal peptides for cellular secretion. They all possess single or even two glyco18 domains with potential or, in the case of Drosophila Cht5, shown chitinolytic activity^40^. Because Cht7 contains a transmembrane domain, it can be assumed that it acts at the apical surface, as shown in other insects^54^. Cht5, Cht6, and Cht12 have domains for binding to chitin, possibly for their specific use within the chitin-matrix. Cht2 has no further domain and is likely not restricted to a specific site within the chitin-matrix. Our analyses show that Cht2 accumulates mainly at the apical cell surface, where the matrix forms in the cuticle assembly zone. To conclude, it is thinkable that the different Chitinases could be responsible for specific steps of chitin modulation within the tracheal chitinous cuticle.

## Discussion

Due to tracheal phenotypes, tracheal expression patterns, and localization data, we show that Cht2, Cht5, Cht6, Cht7, Cht12, and Idgf1, Idgf3, Idgf6 act at the flexible tracheal chitin-cuticle to support tube function. Yet, we do not know their molecular roles at the cuticle. Idgfs contain non-chitinolytic domains^40^, suggesting that they support but do not directly degrade chitin. Chitinases contain different protein domains, suggesting that they can perform specific functions (Fig. S1B). All tracheal chitinases have signal peptides for cellular secretion. They all possess single or even two glyco18 domains with potential or, in the case of *Drosophila* Cht5, shown chitinolytic activity^40^. Because Cht7 contains a transmembrane domain, it can be assumed that it acts at the apical surface, as shown in other insects^54^. Cht5, Cht6, and Cht12 have domains for binding to chitin, possibly for their specific use within the chitin-matrix. Cht2 has no further domain and is likely not restricted to a specific site within the chitin-matrix. Our analyses show that Cht2 accumulates mainly at the apical cell surface, where the matrix forms in the cuticle assembly zone. To conclude, it is thinkable that the different Chitinases could be responsible for specific steps of chitin modulation within the tracheal chitinous cuticle.

### Specific Chitinases form and degrade non-laminar chitin-cuticle

The cuticle protects and shapes insects and their exposed organs. In this context, chitin-matrix plays an integral role. The loss or massive defects of chitin-cuticles lead to the loss of the extracellular barriers against dehydration, pathogenic infections, and mechanical forces. However, the formation of new cuticles often involves the degradation of older chitin that is no longer needed. This is also the case when tubes remove the chitin-cable from their lumina in favor of gas filling. To detect potential organ-specific chitin-cuticle degradation processes, the identification of involved genes and their functional description is of great interest. We assumed that chitinases, which are involved in chitin degradation of the tight epidermal cuticle, might also be needed for the degradation of soft tracheal chitin-cuticle. Our tracheal-specific knockdown studies demonstrate that not all but a specific set of these chitinolytic enzymes and related proteins are required. We found five Chitinases [Cht2,5,6,7,12] and three IDGFs [Idgf1,3,6] to be essential in proper airway gas filling (Fig. 1), which we refer to as tracheal *chitinases* and *idgfs*. It has been shown that gas-filling defects are not lethal to the embryos, they hatch as first instar, and in mild cases can even reach later larval stages^26,31,46,55,56^. Thus, we did not investigate lethality upon tracheal-specific knockdown but found in a previous study that ectodermal 69BGal4 driven knockdown of the five tracheal *chitinases* and three *idgf* genes caused up to 80% lethality during larval stages, which could be caused to some extent by the gas-filling defects^42^.

Further analyses showed that many but not all tracheal Chitinases/Idgfs are involved in the degradation of the chitin-cable. Five genes, namely cht2, cht5, cht6, and idgf3, idgf 6 contained residues of the chitin-cable upon tracheal-specific blt-Gal4-driven knockdown (Fig. 2) indicating their involvement in the degradation of chitin. This result suggests that additional other reasons can be responsible for the defective gas filling. The tracheal specific knockdown of *cht7* can cause clogged smaller branches, without affecting the degradation of the chitin-cable in the dorsal trunk. Reduced levels of the *cht12* and *idgf1* did not show defective tracheal chitin-cable degradation, such as unstable tracheal branches with presumably corresponding barrier defects at tracheal lumina (Fig. S1).

Before airway gas filling takes place in stage 17, tracheal branches need to receive their proper sizes already at stage 16. It is well-known that chitin-cuticle defects severely impact tracheal branch size regulation in late embryos. So far, it was not known whether chitin degradation coincides with chitin-cuticle assembly in earlier stages of tracheal development and if chitinases could be involved in this process. Considering that growing chitin polymers and fibers cannot be endlessly long, the consequence might be that cuticle assembly needs enzymatically ordered processing of chitin polymers. We believe that with the help of chitinases growing chitin polymers can be adapted to local conditions required to assemble the cuticles correctly. We hypothesize that the loss or reduction of chitinase activity could interfere with chitin during the formation of the chitin-matrix. This may not change the placement of cuticular proteins, but it may change the structure and function of chitin within the matrix. Consistently, Cht2 enriched at the apical surface of tracheal cells in stage 16 embryos. Furthermore, our data indicate that altogether three *chitinases [cht2,5,6]* and two *idgfs [idgf3,6]* are involved in the regulation of branch length, which is controlled by the proper chitin-matrix assembly. We focused our studies on identifying the genes involved in tubular size control. However, whether these genes are regulated in their expression and localization patterns by other genes involved in tube size control remains to be investigated. Interestingly, the same genes are involved later on in the degradation of the luminal chitin-cable, suggesting that we have identified the core group of tracheal *chitinases* and *idgfs*.

Thus, both processing and degrading chitin would be consistent with the chitinolytic function of chitinases. Since there could be redundant or overlapping function among the tracheal Chitinases, we cannot exclude that Cht12 is involved in airway clearance, too, and that in case of loss or reduction of more than one *chitinases* phenotypes could become more dramatic. Regardless of this, our Cht2 expression and localization studies in the chitinase knockdown embryos suggest an independent Cht2 function in the tracheal system, although we cannot exclude that knockdown of the genes was not sufficient. The non-chitinolytic *Idgfs* could compete with chitinases for binding to chitin and thus protect chitin from its degradation, which is consistent with the observation that Idgfs, bind colloidal chitin, despite lacking chitin-binding domains^40^. Alternatively, Idgfs could control proper localization of essential chitin-cuticle proteins such as Obst-A, Serp, Verm, and Knk, however, this remains elusive.

Cht2 plays another critical role during tracheal development. The arrangement of the taenidial folds depends primarily on the interaction between endocytosis processes, apical actin formation, and the formation of the chitin-matrix at the apical cell surface. A disturbed taenidial fold arrangement is linked to tube collapse and gas-filling defects^57–59^. Our studies suggest that the formation of taenidial chitin requires Cht2. At reduced Cht2 levels, irregular taenidial folds form, which likely affecting tube stability, which would be another cause of the observed gas filling defects. The increased Cht2 levels degrade chitin excessively on the apical surface, which disrupts the regular taenidial fold pattern.

### Evolutionarily conserved molecules organizing different types of cuticles

Mammals possess no chitin, but since chitin derivatives are biocompatible, they are widely used in medical practice. Nevertheless, because mammals are exposed to chitin and its derivatives, they express chitinases as key degradation enzymes for defense against infections with chitin-containing organisms^37^. Chitin (β-(1–4)-poly-N-acetyl D-glucosamine) itself is widely distributed in nature as a structural component found in bacteria, fungi, parasitic nematodes, and as cuticles from crustaceans to insects. Cuticles consist of highly organized chitin fibers embedded in protein matrices. The chitin-rich part of the cuticle forms highly-ordered, often even laminar structures that are produced at the apical cell surface, in a narrow area called the cuticle assembly zone (Schmidt layer)^12^. We have identified conserved proteins that have critical roles in establishing the non-laminar soft tracheal chitin-matrix. Importantly, these proteins have similarly crucial roles in the epidermal cuticle assembly zone for the formation of the laminar thick chitin-matrix^42,53^. One of these proteins in the epidermis was Cht2, which also plays a prominent role in the assembly of the chitin-matrix in the tracheal system. These results show that very basic processes of chitin cuticle assembly and degradation are molecularly conserved and require a core of critical proteins, such as Cht2.

## Materials and methods

### Fly husbandry, brightfield microscopy, and statistical analysis

We obtained used fly stocks from the Vienna and Bloomington stock centers. We used w^1118^ as control (referred to as wild type, wt) and *breathless(btl)-*Gal4 and 69B-Gal4 as driver lines. Supplemental tables list all used fly lines. Knockdown and control experiments were performed at 28 °C. In general, RNAi induced phenotypes showed variability to some extent form mild to severe appearance. We show representative images that were found in a high number of analyzed embryos and larvae. The number of analyzed animals and statistics of identified phenotypes are indicated in the legends or figures. For gas filling assay, we transferred stage 17 embryos and freshly hatched larvae onto agar plates and studied those by bright field microscopy (Zeiss Axoiplan, A-PLAN 10x/0.25 objective, monochromatic AxioCam). Significance was tested using t-tests in Excel 2019; asterisks indicate p-values (*p < 0.05, **p < 0.01, ***p < 0.001); error bars indicate the standard deviation. Images were processed with ZEN 2.3 and cut with Photoshop CS6.

### Fixation and protein localization studies

Fixation and immunolabeling were performed as recently described^9^. Embryos were bleached for 5 min in sodium-hypochlorite and fixed for 20 min in 4% formaldehyde. After devitellinization (heptane/methanol), we washed and kept samples in methanol for further handling.

For immunolabeling, we washed embryos intensively with PBT (PBS, Tween 20) and with PBT plus 2.5% donkey Serum for 20 min before and after each antibody treatment. Primary antibodies, see supplemental tables, were incubated for 72 h at 4 °C and detected by secondary antibodies, incubated ON. Samples were embedded in Glycerol gelatine (Roth, Karlsruhe). We used secondary antibodies from Molecular probes (Alexa-488, -555, -633, -647; Invitrogen, Carlsbad) and Dianova (Cy2,Cy3,Cy5; JacksonImmuno, Westgrove). Immunostainings were analyzed with Zeiss LSM710 and LSM780-Airysacn (Zeiss, Jena) microscopes, using 25x/0.8 PLAN Neofluar and 63x/1.3 PLAN Neofluar objectives and Zeiss glycerol medium. We used ZEN 2.3 (black) with standard settings for confocal microscopy (Airy 1) and image processing (maximum intensity projection and orthogonal section). We processed indicated confocal images with Huygens Professional deconvolution using the “express” mode (SVI, Netherlands). We cropped Images with Adobe CS6 Photoshop and designed figures with CS6 Illustrator.

For the dorsal trunk measurements, we used confocal Z-stacks of whole-mount embryos of n = 5 embryos for each genotype. In order to compare individual sizes of all different embryos, the measured values (in µm) of individual dorsal trunks were normalized to the corresponding distances between the used endpoints. This normalization led to a better comparison of how strong the tube convolutions are within a defined range. For our analyses, we have used the whole dorsal trunks. Images were taken and measured with ZEN 2.3 (black). All statistics were generated with Excel (2019).

### Whole-mount in situ hybridizations of embryos

According to the manufacturer’s instructions for DIG labeling KIT (Roche), we generated digoxigenin (dig)-labeled RNA sense and antisense probes by in vitro transcription of the cht5 cDNA clone obtained from Drosophila Genomics Resource Center (#1135722; FBcl0236609). Further genes/clones: cht2, LD28264; cht3, LP01426; cht6, IP07037; cht 7, LD 45559; cht12, AT18578; idgf1, RE68533; idgf3, RE62596; idgf6, LD34164. The fixed embryos were washed with PBT and then fixed again for 20 min with PBT: 4% formaldehyde (1:1). We washed embryos with PBT/hybridization buffer (1:1 ratio) three times and finally for 60 min at 60 °C. Hybridization occurred at 60 °C ON. We washed embryos intensively with PBT for standard antibody staining (procedure see above). The dig-labeled RNA was detected with an anti-Dig alkaline phosphatase-conjugated antibody (1:500; Fab fragment; sheep), which we visualized accordingly to the manufacturer’s instructions (Roche).

### Ultrastructure analysis

Third instar larvae were collected from agar plates. Further treatment of larvae and Transmission electron microscopically ultrastructure determinations were performed as described recently^42, 43^.

## Supplementary information

Supplementary file1 (PDF 4535 kb)
