# Supplementary material for: Glycosylhydrolase genes control respiratory tubes sizes and airway stability
Source: Sci Rep. 2020 Aug 7;10:13377. doi: 10.1038/s41598-020-70185-w (PMC7414880; doi:10.1038/s41598-020-70185-w)
Supplement: Supplementary file 1 — Supplementary file1 (PDF 4535 kb) [file 41598_2020_70185_MOESM1_ESM.pdf]

Supplementary Information

Glycosylhydrolase genes control respiratory tubes sizes and airway stability

Matthias Behr[a*], Dietmar Riedel[b]

[a]Institute for Biology, Leipzig University, Philipp-Rosenthal-Str. 55, 04103 Leipzig, Germany

[b]Max Planck Institute for Biophysical Chemistry, Electron microscopy group, 37077 Göttingen, Germany

*Correspondence: matthias.behr@uni-leipzig.de

Supplementary Figure S1

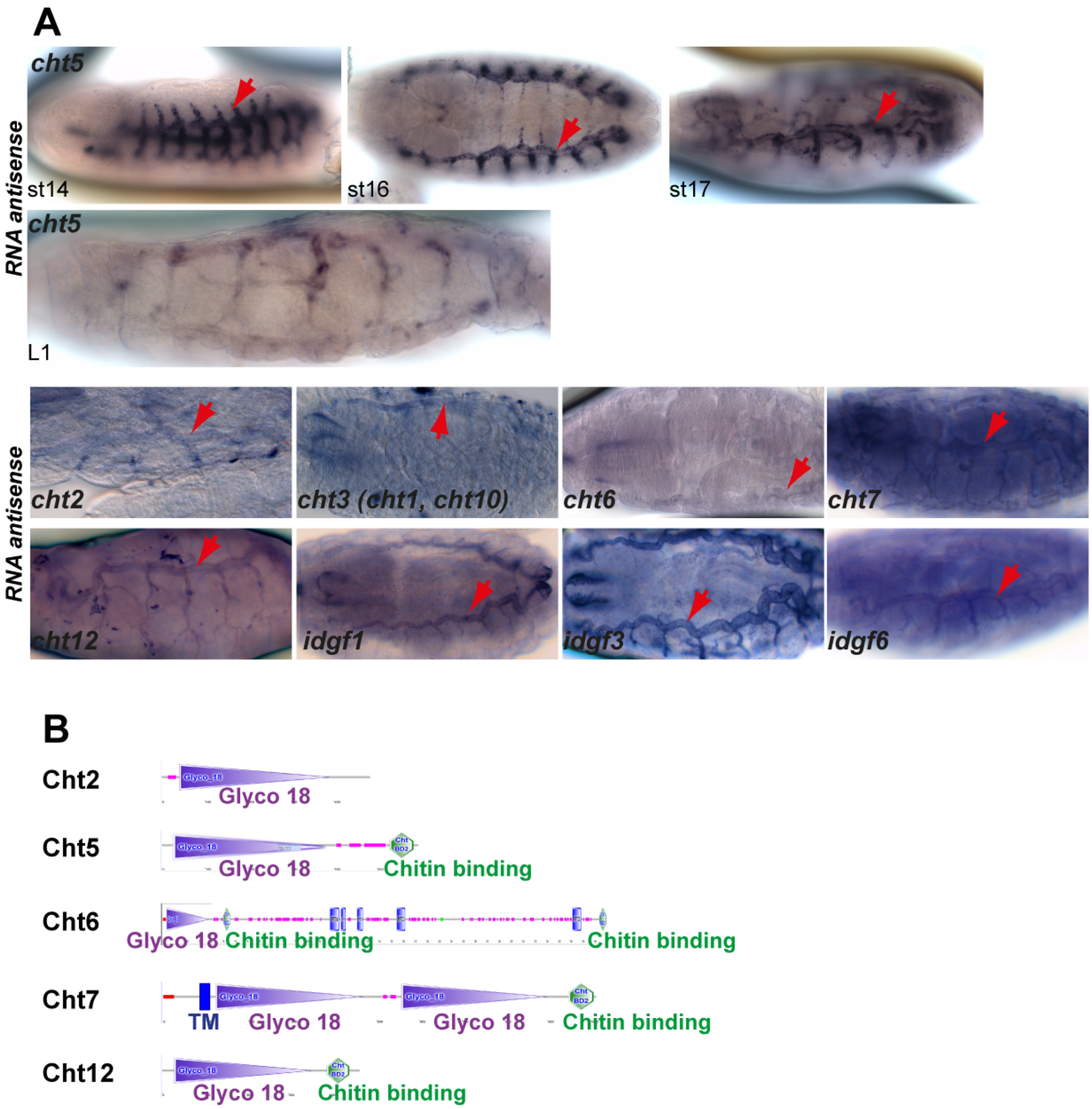

Gene expression and diversity of domain arrangement of tracheal Chitinases. (A) Whole-mount RNA in situ hybridization studies showing expression of the *cht5* gene in tracheal cells (red arrows) of embryos and first instar larva (L1). Whole-mount RNA in situ hybridization of embryos showing expression of the *cht2*, *cht3 (cht1 and cht10)*, *cht6*, *cht7*, *cht12*, *idgf1*, *idgf3*, *idgf6* genes in tracheal cells (red arrows). **(B)** We used the SMART [1] sequence tool to analyze the domain arrangement of tracheal Chitinases. They possess single (Cht2,5,6,12) or two (Cht7) glyco18 domains (purple); N-terminal-signal-peptides (red; previous studies identified a unique N-terminal signal peptide for each chitinase); chitin-binding domains type 2 (Cht5,6,7,12; green hexagon), transmembrane domain (Cht7, blue bar), and internal repeats (Cht6, blue boxes). The individual sizes are not aligned between the Chitinases.

Supplementary Figure S2

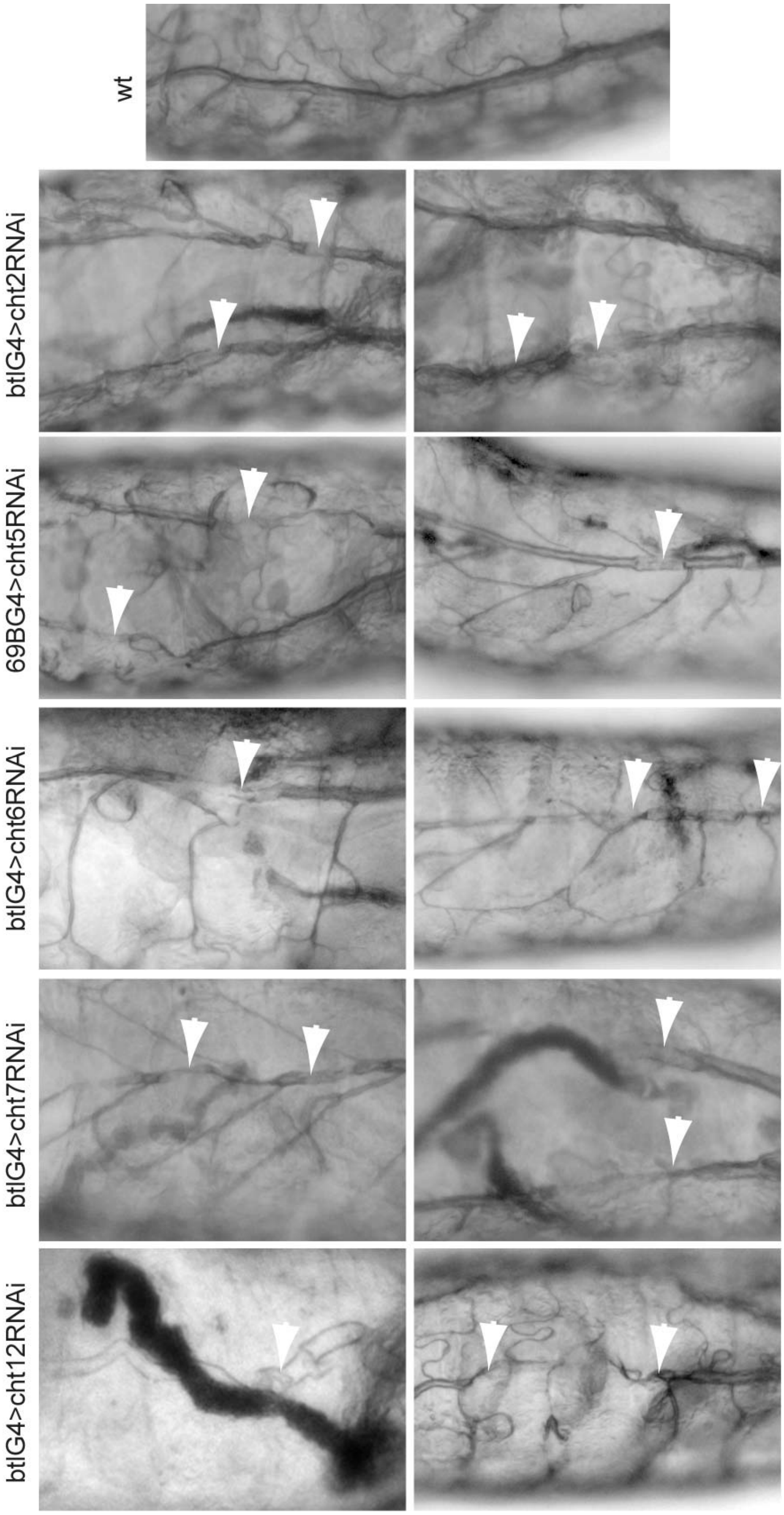

Tracheal chitinases support tube stability and integrity. Impairment of tracheal *chitinases* caused tracheal gas-filling defects and tracheal tube damage (arrows) in first instar larvae. *In vivo* brightfield microscopy analysis of whole-mount first instar larvae. Magnifications focusing on the dorsal trunks show continuous tubes in wt larvae. In contrast, upon knockdown of tracheal *chitinases* (*cht2,5,6,7,12*) tubes appeared to be twisted, crushed, or discontinuous.

Supplementary Figure S3

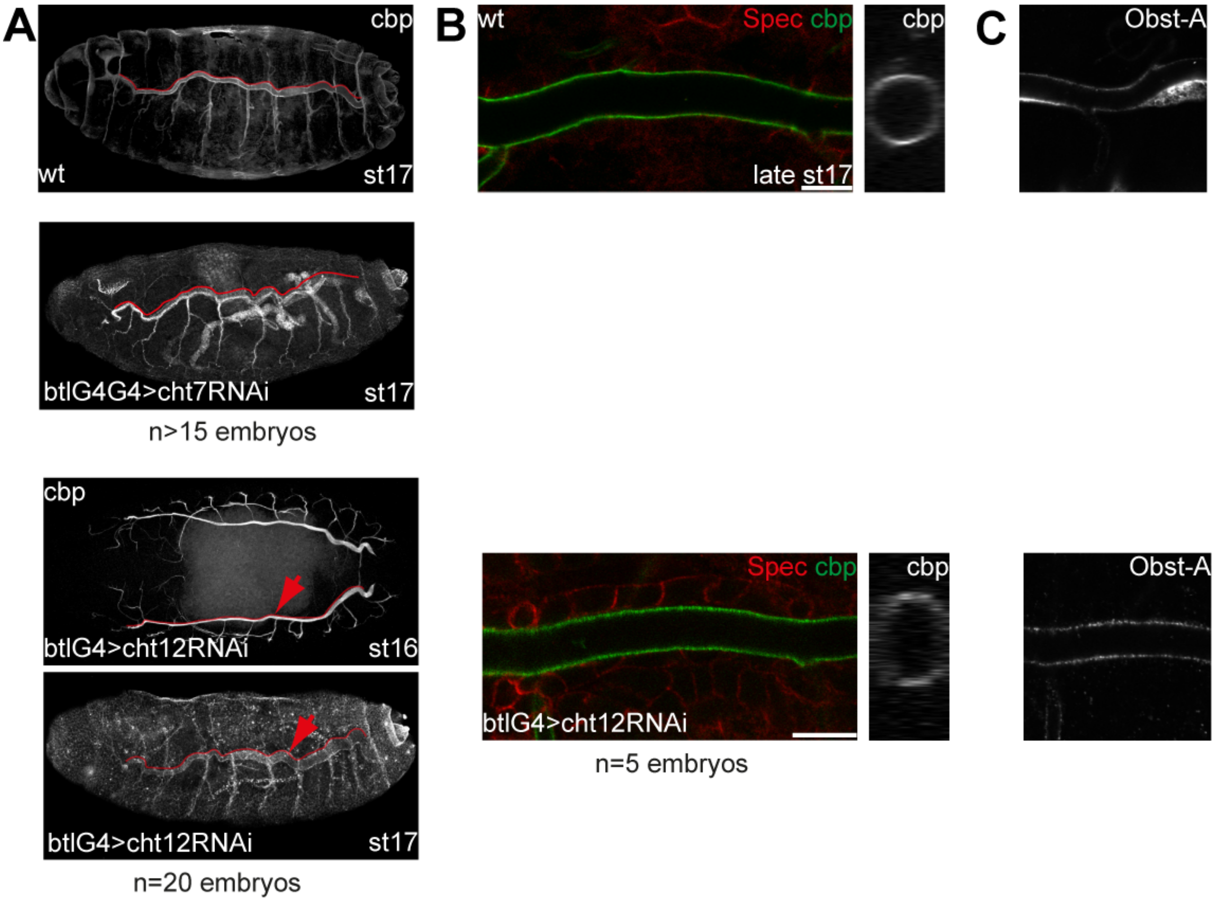

Embryonic tracheal tube size and airway clearance. (A) Upon knockdown *cht7* and *cht12* tracheal dorsal trunks (red lines) showed wt-like appearance (arrow in A) in stage 16 and stage 17 embryos (n=20). **(B)** The reduced *cht12* RNA showed wt-like airway clearance at the end of embryogenesis (n=5), as demonstrated by confocal images and orthogonal projections (left panel). **(C)** Tracheal *cht12* knockdown showed a wt-like apical Obst-A localization in tracheal tubes of stage 17 embryos. Scale bars indicate 10µm.

Supplementary Figure S4

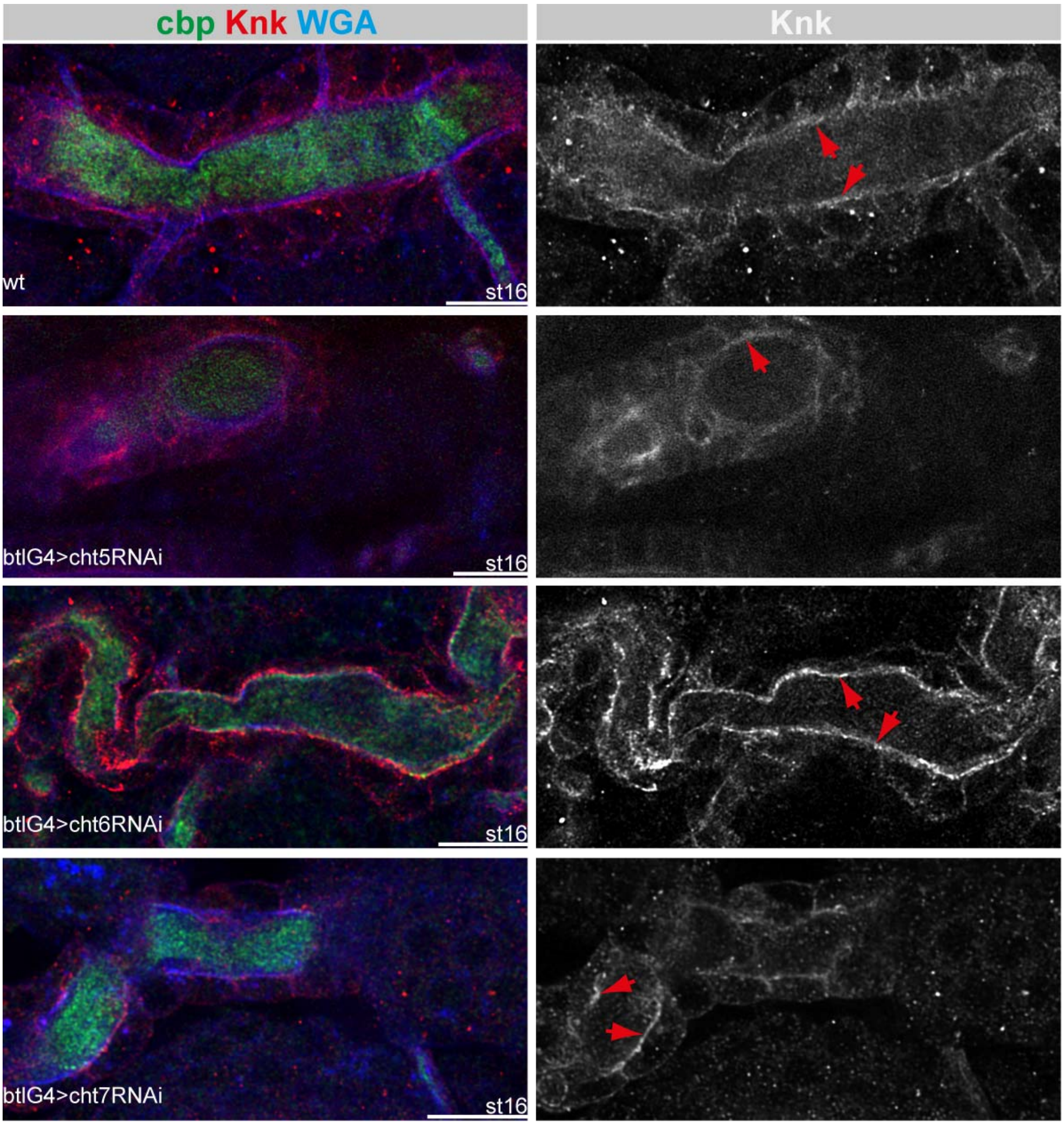

Chitinases do not interfere with Knk localization. In wt cells, Knk (red) localizes apically in the chitin-cuticle of tracheal tubes in stage 16 embryos. cbp (green) detects chitin and WGA (blue) the apical surface. The tracheal specific knockdown of the tracheal *chitinases* (*cht2,5,6,7*) did not disturb the extracellular localization of Knk in the trachea (red arrows point to the apical cell surfaces). We show the tracheal knockdown of *cht2* in Figure 2C. We processed confocal images with Huygens Professional deconvolution using the “express” mode. Scale bars indicate 10µm.

Supplementary Figure S5

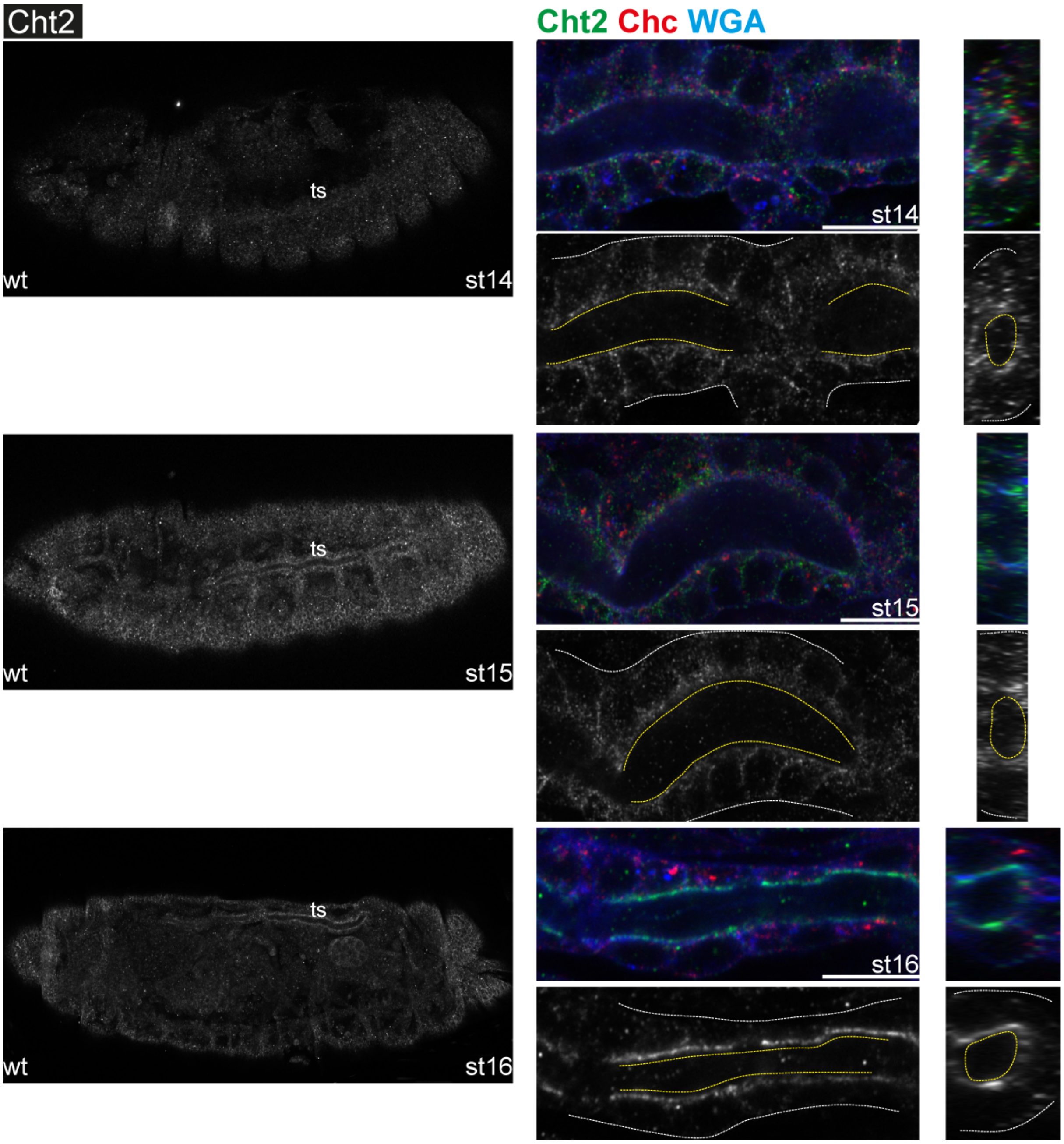

Tracheal Cht2 expression in embryos. **Left panel:** Whole-mount immunofluorescent stainings with the Cht2 antibody (green) of embryos of stages 14, 15, and 16. WGA (blue) detects the apical cell surfaces and chitin, and Clathrin heavy chain (Chc) apical clathrin-vesicles (red); ts, tracheal system. **Middle panel:** Airyscan images focusing on the tracheal dorsal trunk tubes. **Right panel:** orthogonal projections of confocal Z-stacks showing the tube lumen and tracheal cells. White dashes indicate the basal cell site of the tubes, and yellow dashes the apical cell surfaces. In summary, images reveal that Cht2 expression starts at stage 14 in tracheal cells. Cht2 shows the distribution in tracheal cells of stage 14 and 15 embryos. Later on Cht2 accumulates at the tracheal apical cell surface in stage 16 embryos. Scale bars indicate 10µm.

Supplementary Figure S6

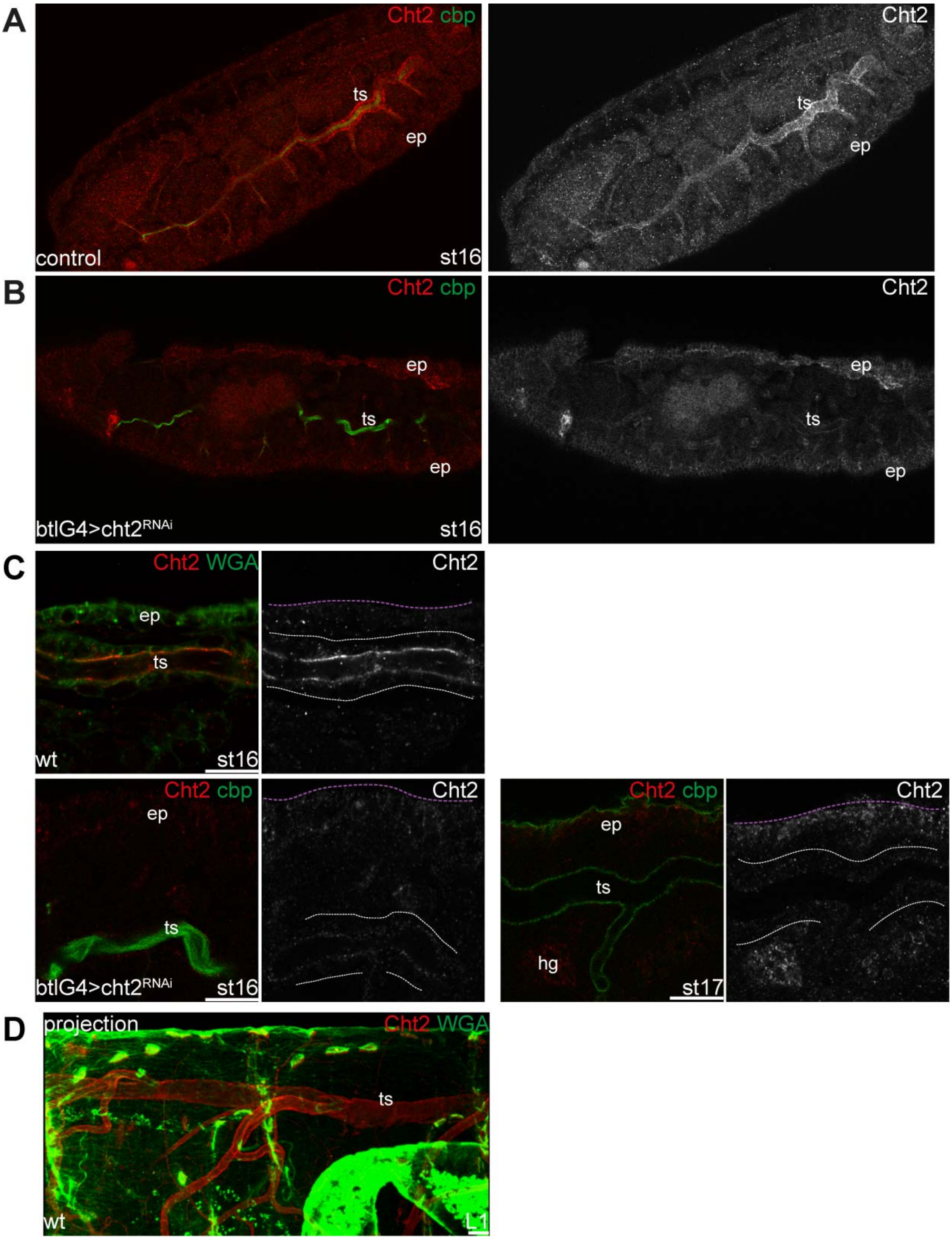

Tracheal Cht2 expression in embryos and larvae. (A) Stage 16 whole-mount immunofluorescent staining with the Cht2 antibody revealed the strongest Cht2 protein expression (red) in the tracheal cells

(tracheal system, ts) and weaker expression in epidermal cells (ep). To detect tracheal chitin, we used cbp (in green). **(B)** Tracheal specific *cht2* knockdown reduced the Cht2 protein expression in the trachea when compared to the epidermis. Reduction of Cht2 protein levels are in agreement with diminished *cht2* RNA levels upon ubiquitous *cht2* knockdown[2]. Note that chitin levels appeared wt-like in *cht2* knockdown embryos, arguing that Cht2 does not affect chitin levels at embryonic stage 16, when chitin-matrix assembles. **(C)** Confocal images are focusing on the tracheal dorsal trunk tubes. In stage 16 and stage 17 wt embryos, Cht2 accumulated at the apical cell surface (see also Figs. 4D; 5A,D; Supplementary Figure S5). Upon tracheal specific *cht2* knockdown, we detected strongly reduced Cht2 protein staining in tracheal cells and at their apical cell surfaces. These findings are supported by the Cht2 stainings of the neighboring epidermis. While epidermal Cht2 staining is detectable, tracheal Cht2 staining appeared reduced upon the tracheal-specific *cht2* knockdown embryos. White dashes mark the basal site of the tracheal tubes, and purple dashes indicate the epidermal apical cell surface; ep, epidermis, ts, tracheal system. Scale bars 10µm. **(D)** In the first instar wt larvae, Cht2 revealed strong expression in the trachea and epidermis. Recently we have demonstrated the specificity of the Cht2 antibody in embryos and larvae [3].

Supplementary Figure S7

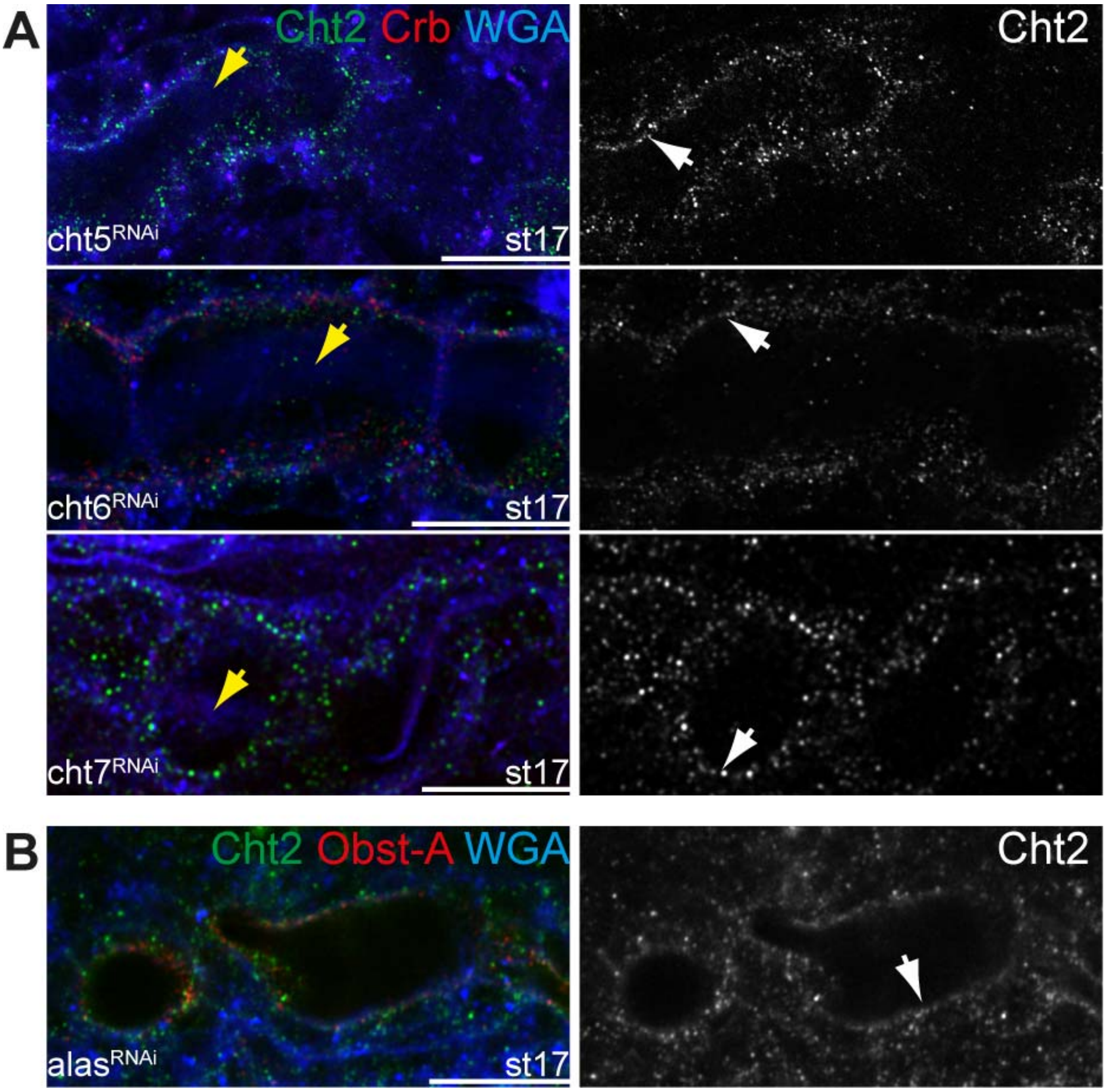

Tracheal Cht2 localization at the end of embryogenesis. **(A)** Immunofluorescent stainings of Cht2 (green), Crb (red) and WGA in stage 17 embryos. The tracheal specific knockdown of *cht5,6,7* revealed wt-like apical Cht2 localization (arrows) at the end of embryogenesis. The knockdown embryos do not show Cht2 accumulation in the residual chitin-cable (yellow arrows). In addition, *alas* knockdown revealed wt-like apical Cht2 localization (arrow) in stage 17 embryos. Scale bars represent 10µm.

Supplementary Table

Resources used for the experimental analysis [3–8]

Gene	Synonyms	RNAi stock	on, off target	embryo expression	off target
cht2	CG2054	111252/KK	1, 1	cuticle organs	CG15899, non-tracheal
cht5	CG9307	109467/KK	1, 1	trachea	Cht11, non-tracheal
cht6	CG43374	102293/KK	1, 0	unknown	no
cht7	CG1869	102683/KK	1, 0	cuticle organs	no
cht12	CG30293	108476/KK	1, 1	cuticle organs	Cht8, non-tracheal
cht1,3,10	CG18140	not analyzed	-	cuticle organs	-
cht8	CG9357	106712/KK	1, 0	no expression	no
cht9	CG10531	16071/GD	1, 0	no expression	no
cht11	CG3044	3262/GD	1, 2	no expression	Cht5, twister

Gene	Synonyms	RNAi stock	on, off target	embryo expression	off target
idgf1	CG4472	12414/GD	1, 1	cuticle organs	idgf4, non-tracheal
idgf3	CG4559	12423/GD	1, 0	cuticle organs & gut	no
idgf6	CG5210	100906/KK	1, 0	cuticle organs & gut	no
idgf2	CG4475	not analyzed	-	gut and fat body	idgf4, non-tracheal
idgf4	CG1780	104968/KK	1, 0	gut and fat body	no
idgf5	CG5154	10097/KK	1,1	fat body	idgf3

reagent	dilution	host animal	origin
**anti-Cht2**	1:40	guinea pig, rabbit	Pesch et al., 2017
**anti-Chc**	1:40	rat	Wingen et al., 2009
**anti-DIG-AP**	1:500	sheep	Roche
**anti-Knk**	1:100	rabbit	Moussian et al., 2006
**anti-Mega**	1:50	mouse	Jaspers et al., 2012
**anti-Obst-A**	1:50	rabbit	Petkau et al., 2012
**anti-α-Spectrin (3A9)**	1:10	mouse	DSHB
**anti-Verm**	1:75	rabbit	Luschnig et al., 2006
**Anti-Wurst**	1:40	guinea pig	Stümpges & Behr, 2011
**CBP – Alexa488**	1:100		Pesch et al., 2015
**WGA – Alexa 633**	1:150		Molecular Probes

Fly lines	origin	Expression pattern
**UAS-alas-RNAi**	Shaik et al., 2012	
**mega^{G0012}**	Behr et al.,, 2003	
**obst-A^{d03}**	Petkau et al., 2012	
**serp,verm**	Luschnig et al., 2006	
**UAS-shibire^{k44} (shi^{DN})**	#5811 Bloom. Stock center	
**btl-G4**	Shiga et al., 1996	trachea, ventral midline
**69B-Gal4**	Brand & Perrimon, 1993	trachea, and other ectodermal organs

Supplementary References

1. Letunic, I. & Bork, P. 20 years of the SMART protein domain annotation resource. *Nucleic Acids Research* **46,** D493-D496; 10.1093/nar/gkx922 (2018).

2. Pesch, Y.-Y., Riedel, D., Patil, K. R., Loch, G. & Behr, M. Chitinases and Imaginal disc growth factors organize the extracellular matrix formation at barrier tissues in insects. *Sci. Rep.* **6,** 18340; 10.1038/srep18340 (2016).

3. Pesch, Y.-Y., Riedel, D. & Behr, M. Drosophila Chitinase 2 is expressed in chitin producing organs for cuticle formation. *Arthropod Structure & Development* **46,** 4–12; 10.1016/j.asd.2016.11.002 (2017).

4. Jaspers, M. H. J. *et al.* The claudin Megatrachea protein complex. *J. Biol. Chem.* **287,** 36756–36765; 10.1074/jbc.M112.399410 (2012).

5. Moussian, B. *et al.* Drosophila Knickkopf and Retroactive are needed for epithelial tube growth and cuticle differentiation through their specific requirement for chitin filament organization. *Development (Cambridge, England)* **133,** 163–171; 10.1242/dev.02177 (2006).

6. Petkau, G., Wingen, C., Jussen, L. C. A., Radtke, T. & Behr, M. Obstructor-A Is Required for Epithelial Extracellular Matrix Dynamics, Exoskeleton Function, and Tubulogenesis. *J. Biol. Chem.* **287,** 21396–21405; 10.1074/jbc.M112.359984 (2012).

7. Luschnig, S., Bätz, T., Armbruster, K. & Krasnow, M. A. serpentine and vermiform Encode Matrix Proteins with Chitin Binding and Deacetylation Domains that Limit Tracheal Tube Length in Drosophila. *Current Biology* **16,** 186–194; 10.1016/j.cub.2005.11.072 (2006).

8. Pesch, Y.-Y., Riedel, D. & Behr, M. Obstructor A Organizes Matrix Assembly at the Apical Cell Surface to Promote Enzymatic Cuticle Maturation in Drosophila. *J. Biol. Chem.* **290,** 10071–10082; 10.1074/jbc.M114.614933 (2015).
